# ER stress sensing mechanism: Putting off the brake on UPR transducers

**DOI:** 10.18632/oncotarget.25114

**Published:** 2018-04-13

**Authors:** Diego Rojas-Rivera, Diego A. Rodriguez, Denisse Sepulveda, Claudio Hetz

**Affiliations:** Biomedical Neuroscience Institute (BNI), Faculty of Medicine, University of Chile, Santiago, Chile; Center for Geroscience, Brain Health, and Metabolism (GERO), Santiago, Chile; Program of Cellular and Molecular Biology, Institute of Biomedical Sciences, University of Chile, Santiago, Chile; Buck Institute for Research on Aging, Novato, CA, USA; Department of Immunology and Infectious Diseases, Harvard School of Public Health, Boston, MA, USA

**Keywords:** Hsp47, IRE1α, UPR, ER stress, BiP, Autophagy

Endoplasmic reticulum (ER) stress is a major contributor to cancer, metabolic disorders and neurodegenerative diseases. ER proteostasis maintenance is controlled by a dynamic signaling network known as the unfolded protein response (UPR). The mechanisms underlying the detection of a stressful condition at the ER are poorly understood and may involve the participation of ER chaperones and the direct recognition of misfolded proteins by specialized sensors. IRE1α is an ER-localized kinase and endoribonuclease that initiates the most conserved UPR signaling branch [1]. IRE1α catalyzes the unconventional splicing of the mRNA encoding the X-box binding protein 1 (XBP1), leading to the expression of an active transcription factor termed XBP1s. In addition, IRE1α regulates the stability of certain mRNAs and miRNAs through a process termed regulated IRE1α- dependent decay (RIDD) [1]. Since ER stress is emerging as a driver of multiple human disorders, a complex network of regulatory checkpoints has evolved to tightly control its signaling behavior. We recently developed a systematic study to identify IRE1α binding partners and discovered a novel ER factor that is necessary for optimal UPR signaling ([2] and see below).

IRE1α is regulated by the assembly of a dynamic protein platform referred to as the UPRosome, which may control the stress threshold to engage the UPR, its temporal inactivation and the cross talk with other stress pathways [3]. How is IRE1α activated? Currently, two models are under debate: a direct recognition model where IRE1α operates as a stress sensor that binds misfolded proteins, and an indirect mechanism where IRE1α transduces stress signals coupled to ER chaperones. An early report indicated that the ER chaperone BiP binds to IRE1α to maintain its monomeric inactive state, an interaction that is lost under stress [4]. Elegant *in vitro* studies demonstrated that the ATPase domain of BiP allosterically associates and represses IRE1α (independent of ATP), whereas the binding of misfolded proteins to the substrate binding domain of BiP triggers the release of IRE1α [5]. A recent report added another piece to the puzzle by identifying the co-chaperone ERdj4 as a possible interface between IRE1α and BiP to repress the pathway [6]. Alternatively, other studies have suggested that misfolded proteins can associate with the luminal domain of IRE1α, allosterically inducing its activation [7].

**Figure 1:**
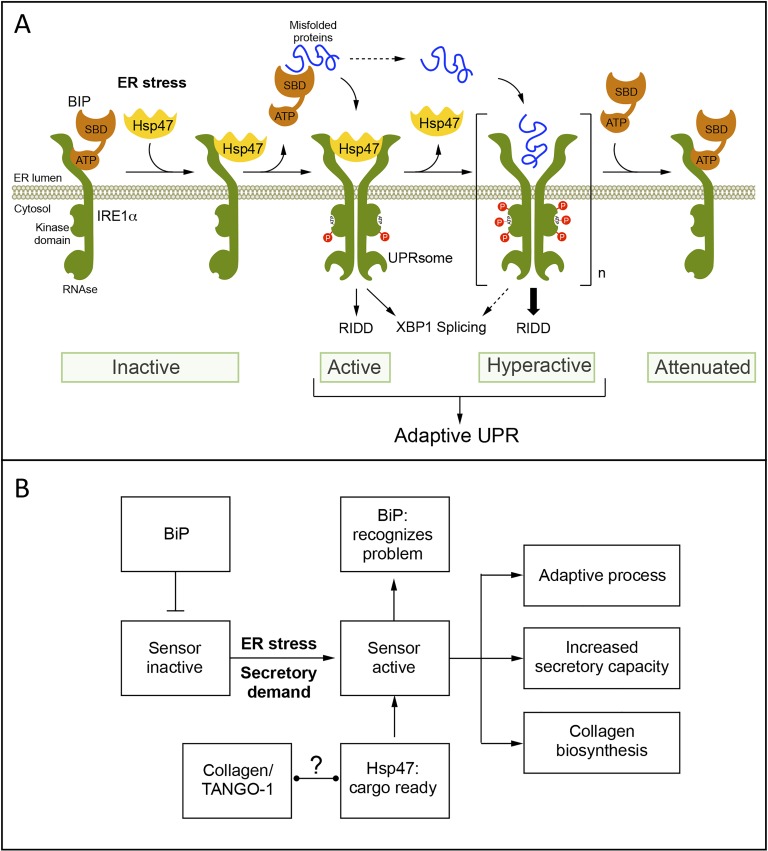
**A.** Working model. Under basal conditions monomeric IRE1α is maintained in an “Inactive” state by directly binding to ER the chaperone BiP via its ATPase domain (ATP). The ER factor Hsp47 transiently binds to IRE1α, promoting the release of BiP from the complex. BiP then associates with misfolded proteins through its substrate binding domain (SBD). These molecular events trigger IRE1α dimerization, autophosphorylation and oligomerization to catalize *Xbp1* mRNA splicing and RIDD. After sustained ER stress, Hsp47 is released from the UPRosome complex. Finally, when ER stress is diminished by the UPR, BiP binds to IRE1α promoting its inactivation. **B.** To factors that regulate the UPR: under stress triggering IRE1α (alpha) activation. In contrast, Hsp47 is involved in the recognition of folded collagens for their export to the Golgi together with TANGO-1. Activation of the UPR not only triggers adaptive processes to mitigate ER stress and increase secretory cell function, but also enforces collagen biosynthesis and secretion, the main client of the ER.

The amplitude and kinetics of ER stress signaling are regulated by the binding of different cofactors to the main UPR transducers [1], however no systematic studies were available to define the nature of the IRE1α interactome. Using a proteomic screening, followed by functional validation, we unveiled Hsp47 as a novel regulator of the UPR transducer IRE1α. Cellular and biochemical characterization indicated that Hsp47 engages IRE1α signaling through a physical interaction, involving the release of BiP from the complex [2]. The control of IRE1α signaling by Hsp47 is evolutionary conserved as validated using *D. melanogaster* and mouse models of ER stress [2]. We propose that Hsp47 is part of a chaperone network that adjusts IRE1α signaling to set the stress threshold of activation to engage the UPR (Figure 1A).

Hsp47 is a specialized cofactor for collagen biosynthesis, the most abundant protein in the cell and the main ER cargo. Hsp47, together with TANGO-1, guide the trafficking of the collagen triple helix into the Golgi apparatus for further maturation and secretion into the extracellular space. Thus, we speculate that the molecular connection identified here between Hsp47 and IRE1α may represent a central mechanism that adjusts the ER folding needs according to the production of collagen, where more collagen expression engages stronger IRE1α signals to improve the secretory capacity of the cell. Our study suggests that there are two types of signals to engage IRE1α: (i) abnormal misfolded proteins that de-repress the transducer through the binding to BiP and (ii) the increased production of properly folded collagens/Hsp47 complexes (Figure 1B). Interestingly, XBP1s was recently shown to control TANGO-1, and *Collagen-6a* is also a major RIDD substrate, suggesting that maintaining an equilibrated pathway to fold collagen is a major challenge to the UPR. Moreover, recent studies in medaka fish indicated that most of the abnormal phenotypes triggered by the genetic disruption of major UPR components are due to the accumulation of misfolded collagens (see all references in [2]). Overall, we propose that the identification of Hsp47 as a positive regulator of IRE1α may reveal a tight relationship between collagen synthesis and UPR activation as a feedback mechanism to cope with the synthesis and secretion of these complex cargoes. Since collagens are the main ER clients, and possibly the main problem under ER stress, our current study may contribute to our understanding of how fluctuations in the ER folding capacity are monitored and adjusted to maintain proteostasis and its relation to pathological conditions such as fibrosis.
